# C-reactive protein to albumin ratio predicts the outcome in renal cell carcinoma: A meta-analysis

**DOI:** 10.1371/journal.pone.0224266

**Published:** 2019-10-23

**Authors:** Wei Zhou, Guang-lin Zhang

**Affiliations:** 1 Department of Urology, Huangshi Central Hospital (Pu Ai Hospital), Affiliated Hospital of Hubei Polytechnic University, Edong Healthcare Group, Huangshi, Hubei,China; 2 Department of Abdominal and Pelvic Medical Oncology II ward, Huangshi Central Hospital (Pu Ai Hospital), Affiliated Hospital of Hubei Polytechnic University, Edong Healthcare Group, Huangshi, Hubei, China; Roswell Park Cancer Institute, UNITED STATES

## Abstract

**Background:**

Growing evidence has revealed that pretreatment C-reactive protein to albumin ratio (CAR) are associated with prognosis for patients with renal cell carcinoma (RCC). However, inconsistent findings have been reported, which promote us to summarize the global predicting role of CAR for survival in RCC patients.

**Methods:**

Two reviewers independently retrieved the literature on EMBASE, MEDLINE, and Cochrane Library databases for eligible studies evaluating the associations of CAR with survival. Data related to the overall survival (OS), disease-free survival (DFS), progress-free survival (PFS), and clinicopathological features were extracted and pooled using meta-analysis with fixed or random- effect models when applicable.

**Results:**

Eight studies including 2,829 patients were analyzed in the present study. High pretreatment CAR was associated with worse OS (pooled HR: 2.14, 95% CI = 1.64–2.79, p < 0.001) and DFS/PFS (pooled HR: 1.75, 95% CI: 1.31–2.35, P < 0.001). Moreover, high CAR was correlated with performance status (≥ 1), tumor location (left), Fuhrman grade (3–4), TNM stage (III-IV), T stage (T3-4), N stage (N1), M stage (M1), tumor necrosis (yes), venous thrombus (positive), metastasis at diagnosis (yes), NLR (> median), and PLR (> median).

**Conclusion:**

High pretreatment CAR is effectively predictive of worse survival in patients with RCC and could be a prognostic biomarker for those patients.

## Introduction

Renal cell carcinoma (RCC) is the most lethal urologic malignancy with incidence rates increase approximately 2% annually [1]. Despite recent efforts in multimodal approaches, RCC remains a huge health burden worldwide and a major cause of mortality due to the high frequent metastasis and recurrence after surgery [2, 3]. At present, there is no effective biomarker for early detection, diagnosis and prognosis of renal tumors. Therefore, there is an urgent need to find a reliable biomarker of RCC to individualized treatment.

It has been reported that cancer-associated inflammation can promote cancer development and angiogenesis [4]. Several inflammatory markers, such as modified Glasgow Prognostic Score (mGPS), C-reactive protein (CRP), and the combination of neutrophil, lymphocyte, monocyte count plays a key role in prognosis in RCC [5–7]. Recently, the C-reactive protein to albumin ratio (CAR) as a novel inflammation-based prognostic score, combination of CRP and albumin, has shown significant prognostic value in RCC [8–10]. However, most of these studies include only small study populations and their conclusions remain inconclusive [11, 12]. The inconsistent findings prompted us to perform this study to provide a comprehensive overview of all reported clinical studies investigating the impact of CAR on prognosis and clinicopathological feature of RCC patients.

## Materials and methods

### Search strategy

The present study was conducted and reported under the guidelines formulated in Preferred Reporting Items for Systematic Reviews and Meta-analyses. A comprehensive literature search was carried out on the basis of the electronic databases including MEDLINE, Embase, and Cochrane Library databases. The literature search was conducted up to June 2019. The key words used included: (“C-reactive protein to Albumin ratio” or “C-reactive protein-to-Albumin ratio” or “C-reactive protein Albumin ratio” or “C-reactive protein/Albumin ratio” or “CRP/Alb ratio”) and (“renal” or “kidney” or “nephron*”) and (“carcinoma” or “cancer” or “tumor” or “neoplasms” or “cancer”). Detailed search strategies refer to S1 Text.

### Inclusion and exclusion criteria

The studies qualified to be included had to meet the following criteria: (1) studies investigating the relationship between pretreatment CAR and RCC prognosis; (2) patients did not receive any treatment (such as surgery or chemotherapy) before obtaining samples; and (3) the study directly provided HRs with 95% CIs or exhibited adequate data which can be used to calculate these statistics. The studies were excluded according to exclusion criteria: (1) duplicated studies, (2) studies provided inadequate survival data for further quantification, and (3) conference abstracts, letters, or case reports.

### Data extraction

Data were extracted using pre-designed standardized forms as following: study characteristics (first author’s name, publication year, region, and sample size); patients information (gender and age, performance status), pathological characteristics (tumor location, histology type, Fuhrman grade, TNM stage, tumor necrosis, venous thrombus, and metastasis at diagnosis), and clinical features (symptoms, type of treatment applied, CAR cut-off values, neutrophil-lymphocyte ratio (NLR), platelet-lymphocyte ratio (PLR), patient’s survival outcome, and follow-up period).

### Quality assessment

The quality assessment of enrolled study was conducted following the guidelines of the Newcastle-Ottawa Scale (NOS), which assessed studies with 9 items including selection, comparability, outcome of interest, follow-up et al [13]. Studies with NOS values greater than 6 are considered high quality studies.

### Statistical analysis

We combined HRs with their corresponding 95% CIs from each eligible study to evaluate the prognostic value of pretreatment CAR in RCC patients. As for clinical features, ORs and associated 95% CI were used. In this meta-analysis, the HRs and 95% CIs were directly extracted if a study reported the survival analysis, otherwise, they were computed from the Kaplan-Meier graph using the software of Engauge Digitizer (version 4.1) [14, 15]. The heterogeneity was tested with Cochran’s Q test and Higgins’s I^2^ statistic. For the presence of heterogeneity (P < 0.05 or/and I^2^ > 50%), a random-effect model was employed to calculate the pooled HRs; otherwise, a fixed effect model was selected (P > 0.05 or/and I^2^ < 50%) [16]. Potential sources of heterogeneity were identified by performing subgroup and sensitivity analyses. All statistical analyses were conducted using Review Manager 5.3 software (Cochrane Collaboration, Copenhagen, Denmark).

## Results

### Included literature

**Fig 1:**
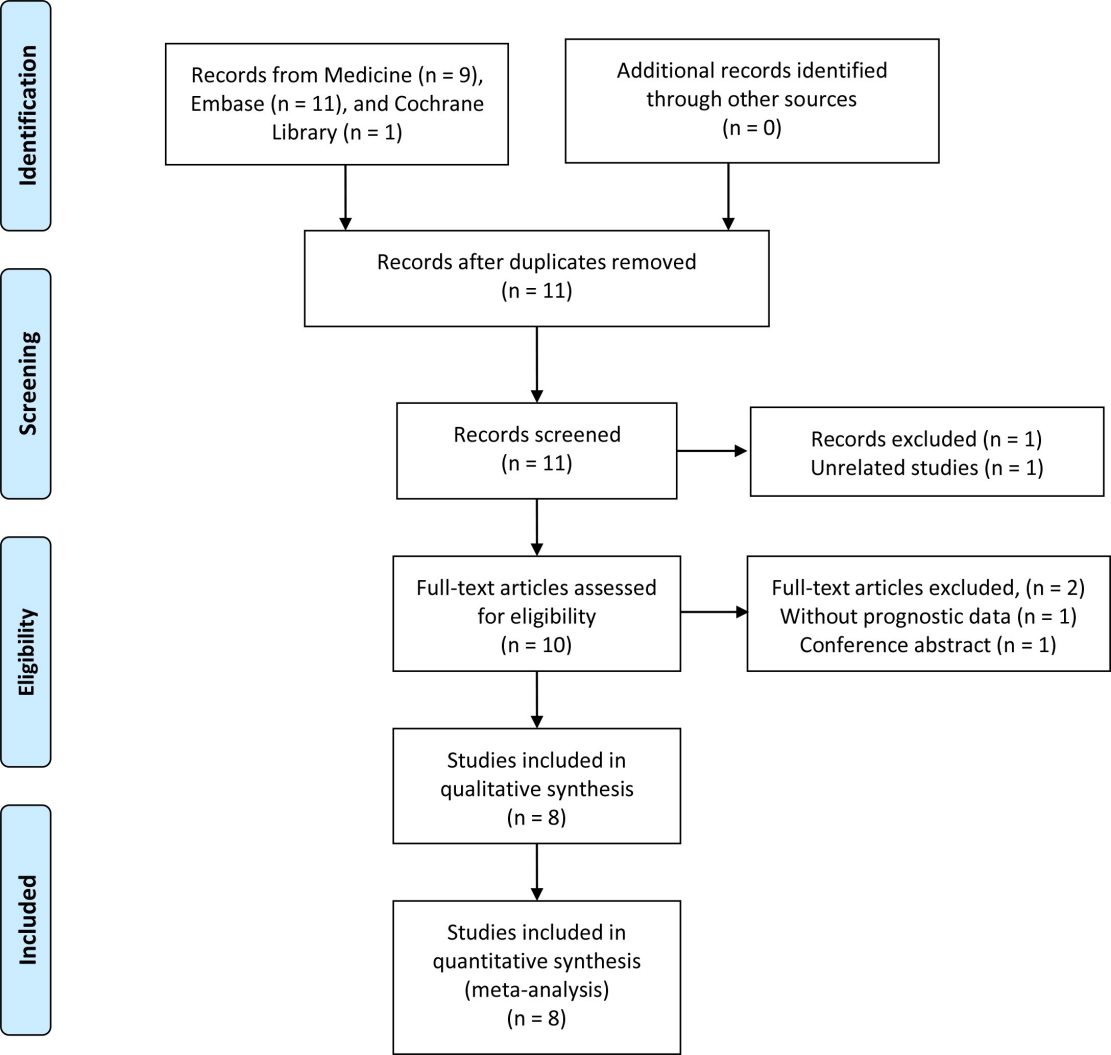
Flowchart describing the literature search and study selection.

Literature research identified 21 records, including 9 from Medline, 11 from Embase, and 1 from Cochrane Library. As shown in the flow diagram for the literature (Fig 1), 11 articles were left after removing duplications. After screening titles and abstracts, 10 full-text articles remained for further assessment. Two articles were excluded according to the inclusion criteria. A total of 8 articles were finally enrolled for the evidence synthesis [8–12, 17–19].

### Study characteristics

The main features of all the eligible studies are summarized in Table 1. Most of included studies were from China and Japan. There were 6 studies were reported at mixed disease, and 2 studies were reported in metastatic disease. All the eligible studies assessed prognostic value of CAR on OS, and 5 studies for DFS or PFS. Cutoff values of CAR ranged from 0.05 to 1.5. The HR and 95% CI data were evaluated using univariate analysis in 1 study and multivariate analysis in 7 studies. The Newcastle-Ottawa Scale (NOS) score of each study included ranged from 7 to 9, suggesting that the methodological qualities were overall moderate to high.

**Table 1:** Characteristics of the studies included in the meta-analysis.

Author	Year	Country	Histology type	Sample size	Age (M±SD, years)	Treatment	Stage	Cut-off value	Outcome	Analysis	NOS score	Follow-up (months)
Komura	2019	Japan	Mixed	757	62.3 ± 11.7	Surgery	Mixed	1.5	OS	UV	7	80
Konishi	2019	Japan	Mixed	176	67 (59–74)	Molecular- targeted therapy	Metastatic	0.05	OS	MV	7	NA
Guo	2017	China	Mixed	570	51.43 ± 13.52	Surgery	Mixed	0.08	OS/DFS	MV	9	65.19
Tsujino	2019	Japan	Mixed	699	61.9 ± 11.7	Surgery	Mixed	0.073	OS/PFS	MV	8	73
Barua	2019	India	Non-clear	31	NA	Surgery	Metastatic	0.11	OS/PFS	MV	6	16.5±1.45
Gao	2019	China	Mixed	108	57 (23–78)	Surgery	Mixed	0.094	OS/DFS	MV	9	54.5 (7.3–74.2)
Agizamhan	2018	China	Mixed	82	NA	Surgery	Mixed	0.083	OS/DFS	MV	8	31 (2–108)
Chen	2015	China	Clear	406	58 (24–80)	Surgery	Mixed	0.06	OS	MV	8	63 (1–151)

Abbreviations: OS: overall survival; DFS: disease-free survival; RFS: relapse-free survival; PFS: progression-free survival; UV: univariate MV: multivariate; NA: not available.

### CAR and OS in RCC

**Fig 2:**
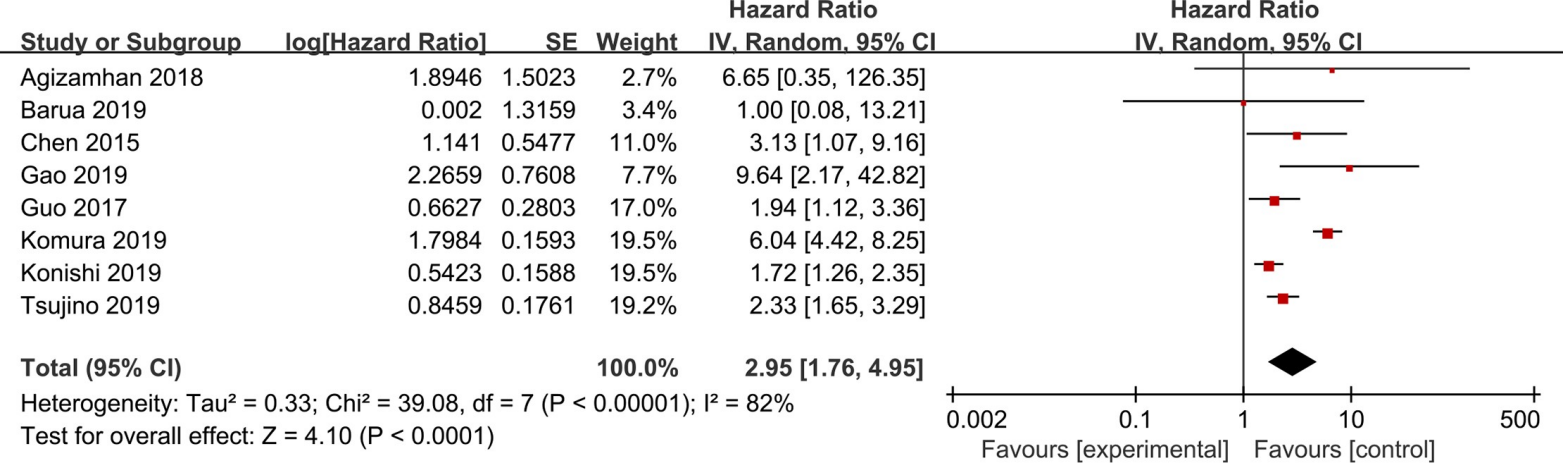
Forest plots of the correlation between CAR and OS in RCC patients.

All included studies including 2,829 patients reported the relationship between CAR and OS in RCC. A random-effects model was applied to estimate the pooled HR and corresponding 95% CI as the significant heterogeneity (I2 = 82%, P < 0.001). As a result, high pretreatment CAR was predictive of a short OS (pooled HR: 2.95, 95% CI: 1.76–4.95, p < 0.001, Fig 2).

To explore the source of heterogeneity, subgroup study was performed (Table 2).

The pooled HRs for all subgroups were not significantly altered by the study characteristics. For example, exploratory subgroup analysis, based on tumor stage, indicated that patients with mixed stage (pooled HR: 3.54; 95% CI = 1.99–6.29; P < 0.001) and metastatic stage (pooled HR: 1.71; 95% CI = 1.25–2.32; P < 0.001) were all significantly associated with worse OS. Similarly, stratified analysis by cut-off for CAR showed that significant poor OSS was observed in both CAR < 0.08 (pooled HR: 2.00; 95% CI = 1.62–2.47; P < 0.001) and patients with CAR > 0.08 (pooled HR: 6.01; 95% CI = 4.45–8.13; P < 0.001). Moreover, histology type, sample size, treatment, and analysis method also did not affect the significant predictive impact of CAR in RCC patients.

**Table 2:** Pooled hazard ratios (HRs) for OS according to subgroup analyses.

Subgroup	No. of studies	No. of patients	HR (95% CI)	P value	Heterogeneity	
					I^2^(%)	Ph
Overall	8	2,829	2.95 (1.76–4.95)	<0.001	82	<0.001
Histology type						
Clear cell carcinoma	1	406	3.13 (1.07–9.13)	0.037	―	―
Others	7	2,423	2.94 (1.67–5.17)	<0.001	85	<0.001
Sample size						
≥ 300	4	2,432	3.09 (1.64–5.80)	<0.001	86	<0.001
< 300	4	397	1.86 (1.37–2.51)	<0.001	49	0.12
Stage						
Mixed	6	2,622	3.54 (1.99–6.29)	<0.001	79	<0.001
Metastatic	2	207	1.71 (1.25–2.32)	<0.001	0	0.68
Cut-off for CAR						
≤ 0.08	4	1,959	2.00 (1.62–2.47)	<0.001	14	0.51
> 0.08	4	870	6.01 (4.45–8.13)	<0.001	0	0.52
Treatment						
Surgery	7	2,653	3.36 (1.92–5.89)	<0.001	75	<0.001
Molecular-targeted therapy	1	176	1.72 (1.26–2.36)	0.001	―	―
Analysis						
Univariate	1	757	6.04 (4.42–8.27)	<0.001	―	―
Multivariate	7	2,072	2.06 (1.68–2.54)	<0.001	19	0.28

### CAR and DFS/PFS in RCC

**Fig 3:**
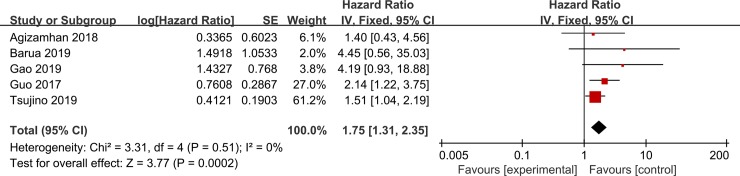
Forest plots of the correlation between CAR and DFS/PFS in RCC patients.

Five studies involving 1,382 patients investigated the correlation between pretreatment CAR and DFS/RFS. According to the final pooled HR of 1.75 (95% CI = 1.31–2.35, P < 0.001, Fig 3), it indicated that high CAR was associated with worse DFS/RFS in patients with RCC.

### CAR and clinicopathological characteristics

A total of 16 variables were investigated in the meta-analysis, including age, gender, performance status, tumor location, symptoms, histology type, Fuhrman grade, TNM stage, T stage, N stage, M stage, tumor necrosis, venous thrombus, metastasis at diagnosis, NLR, and PLR. The results demonstrated that high CAR was related to performance status (≥ 1 vs. 0; OR = 3.86, 95% CI: 2.51–5.95, P < 0.001), tumor location (left vs. right; OR = 1.92, 95% CI: 1.04–3.54, P = 0.04), Fuhrman grade (3–4 vs. 1–2; OR = 3.29, 95% CI: 2.21–4.90, P < 0.001), TNM stage (III-IV vs. I-II; OR = 5.17, 95% CI: 3.58–7.49, P < 0.001), T stage (T3-4 vs. T1-2; OR = 3.46, 95% CI: 1.91–6.25, P < 0.001), N stage (N1 vs. N0; OR = 4.02, 95% CI: 2.77–5.83, P < 0.001), M stage (M1 vs. M0; OR = 12.09, 95% CI: 4.60–31.77, P < 0.001), tumor necrosis (yes vs. no; OR = 2.31, 95% CI: 1.26–4.24, P = 0.007), venous thrombus (positive vs. negative; OR = 16.46, 95% CI: 4.61–58.83, P < 0.001), metastasis at diagnosis (yes vs. no; OR = 3.30, 95% CI: 1.12–9.71, P = 0.03), NLR (> median vs. < median; OR = 2.90, 95% CI: 1.47–5.71, P = 0.002), and PLR (> median vs. < median; OR = 2.92, 95% CI: 1.66–5.15, P < 0.001). However, there was no obvious correlation between CAR and age (> median vs. < median; OR = 1.28, 95% CI: 0.76–2.14, P = 0.36), gender (male vs. female; OR = 1.05, 95% CI: 0.83–1.33, P = 0.70), symptoms (symptomatic vs. asymptomatic; OR = 3.20, 95% CI: 0.67–15.31, P = 0.14), and histology type (clear vs. others; OR = 0.31, 95% CI: 0.07–1.35, P = 0.12). Table 3 lists the details of the relationship between CAR and clinicopathologic parameters.

**Table 3:** Meta-analysis of the association between CAR and clinicopathological features of RCC.

Characteristics	No. of studies	No. of patients	OR (95% CI)	p	Heterogeneity	
					I^2^ (%)	P_h_
Age (> median vs. < median)	4	1,295	1.28 (0.76–2.14)	0.36	70	0.02
Gender (male vs. female)	5	1,342	1.05 (0.83–1.33)	0.70	20	0.29
Performance status (≥ 1 vs. 0)	2	875	3.86 (2.51–5.95)	<0.001	22	0.26
Tumor location (left vs. right)	2	190	1.92 (1.04–3.54)	0.04	0	0.49
Symptoms (symptomatic vs. asymptomatic)	2	190	3.20 (0.67–15.31)	0.14	84	0.01
Histology type (clear vs. others)	3	1,445	0.31 (0.07–1.35)	0.12	95	<0.001
Fuhrman grade (3–4 vs. 1–2)	4	1,010	3.29 (2.21–4.90)	<0.001	42	0.18
TNM Stage (III-IV vs. I-II)	4	1,166	5.17 (3.58–7.49)	<0.001	4	0.37
T stage (T3-4 vs. T1-2)	6	2,041	3.46 (1.91–6.25)	<0.001	76	<0.001
N stage (N1 vs. N0)	6	2,041	4.02 (2.77–5.83)	<0.001	35	0.18
M stage (M1 vs. M0)	4	1,166	12.09 (4.60–31.77)	<0.001	0	0.84
Tumor necrosis (yes vs. no)	2	514	2.31 (1.26–4.24)	0.007	0	0.48
Venous thrombus (positive vs. negative)	2	190	16.46 (4.61–58.83)	<0.001	0	0.74
Metastasis at diagnosis (yes vs. no)	2	875	3.30 (1.12–9.71)	0.03	85	0.01
NLR (> median vs. < median)	2	1,269	2.90 (1.47–5.71)	0.002	84	0.01
PLR (> median vs. < median)	3	1,331	2.92 (1.66–5.15)	<0.001	76	0.02

### Sensitivity analysis

Sensitivity analyses were further carried out to investigate the effect of single study on the overall conclusion. After removing Komura's study, the heterogeneity between studies was significantly reduced (I^2^ = 19%, P = 0.28). However, there is no significant influence on the pooled results of OS (pooled HR: 2.14; 95% CI = 1.64–2.79; P < 0.001), which indicated the robustness of the results described above.

## Discussion

A previous meta-analysis was conducted to evaluate the prognostic value of CAR in patients with variety types of cancer, in which only 2 studies of RCC were included [20]. In addition, they only assessed the prognostic value of CAR in OS without assessing the association between CAR and DFS/PFS and clinicopathological features. To the best of our knowledge, our study is the first and most comprehensive systematic evaluation of the literatures exploring the prognostic impact of pretreatment CAR in RCC survivors. According to the pooled results, there was a significant correlation of high CAR with worse survival of RCC patients, with a combined HR of 2.95 (95% CI 1.76–4.95) for OS, 1.75 (95% CI 1.31–2.35) for DFS/PFS. Subgroup analysis indicated that the predictive efficacy for OS were more significant, regardless of tumor stage, histology type, sample size, treatment, cut-off value for OS, and analysis method. To further explore the source of heterogeneity, we performed sensitive analyses. The results showed that the heterogeneity between the studies was significantly reduced after the removal of Komura’s study. The prognostic value of CAR has not been significantly affected, with HR of 2.14. Moreover, high pretreatment CAR were correlated with advanced clinicopathological characteristics, such as performance status (≥ 1), tumor location (left), Fuhrman grade (3–4), TNM stage (III-IV), T stage (T3-4), N stage (N1), M stage (M1), tumor necrosis (yes), venous thrombus (positive), metastasis at diagnosis (yes), NLR (> median), and PLR (> median). Therefore, CAR provides a potential new prognostic biomarker for cancer control that will help counteract the burden of this disease.

There is a well-documented correlation between the inflammation and cancer, although the exact mechanism is still not fully understood. Inflammatory response can promote tumorigenesis and progression by affecting the tumor microenvironment [21]. Tumor-associated inflammatory response consists of inflammatory cells and a range of inflammatory mediators, such as acute phase proteins, chemokines, and cytokines, which stimulate tumor cell growth, promote angiogenesis, resist cell death and apoptosis, and enhance invasion ability of tumor cells [4, 22]. There is increasing evidence that high levels of systemic inflammatory cells have the potential to serve as prognostic markers in RCC patients. Chen et al. [23] found that patients with high systemic inflammation response index have worse OS and cancer-specific survival (CSS) in RCC. Kim et al. [24] performed a retrospective study with 309 non-metastatic clear cell renal cell carcinoma patients, found that elevated NLR and PLR are indicative of a poor RFS.

Accumulating evidence has indicated that nutrition status and systemic inflammation are involved in tumor progression [8]. Serum CRP and albumin are indicators of chronic inflammation and poor nutritional status of cancer patients [25, 26]. The CAR calculated from the serum CRP and albumin levels. It was originally studied as a prognostic marker for patients with sepsis [27] and was later used as a marker for patients with tumors [28]. Recently, CAR has been reported to predict oncological outcomes in patients with RCC. However, the exact mechanism regarding its prognostic ability have not been clearly elaborated. CRP is an acute-phase protein that is synthesized in the liver, together with cytokines such as interleukin (IL)-1, IL-6, and tumor necrosis factor α [29, 30]. Research has discovered that CRP produces inflammatory cytokines and chemokines, which lead to cancer progression [31]. Several studies have shown that high CRP level was linked to worse survival of RCC patients [32, 33]. Serum albumin is an objective indicator of nutritional status and clinical inflammation that is downregulated in inflammation [34]. Since both proteins are synthesized in hepatocytes, the combination of up-regulated acute phase inflammatory protein and down-regulated chronic phase inflammatory protein may be effective in predicting prognosis.

Several limitations of this study should be considered. First, most of included studies were carried out in Asia. Hence, it is possible that our findings may not extend to other populations across the world. Second, there is a lack of unified cut-off values of CAR. An appropriate definition of the cut-off values is for increased improve survival risk. To a large extent, inconsistencies in methodologies have led to differences in contemporary findings on the prognostic value of CAR. Therefore, determining the standard cut-off value of CAR will significantly promote a final consensus on the prognostic value of CAR. Third, when performing multivariate analysis, the risk factors for adjustment are not exactly the same. Finally, all included studies were retrospective studies.

## Conclusions

Our study demonstrated that pretreatment CAR is significant determinants of shorter OS, DFS, and PFS in patients with RCC.

## Supporting information

S1 Table PRISMA checklist. Completed checklist of PRSIMA guidelines.
(DOC)

S2 Table Study characteristics. Characteristics of the studies included in the meta-analysis.
(XLSX)

S1 Text Search strategies. Search strategy used in meta-analysis.
(DOC)
