# Supplementary material for: C-reactive protein to albumin ratio predicts the outcome in renal cell carcinoma: A meta-analysis
Source: PLoS One. 2019 Oct 23;14(10):e0224266. doi: 10.1371/journal.pone.0224266 (PMC6808556; doi:10.1371/journal.pone.0224266)
Supplement: S1 Text — Search strategy used in meta-analysis. (DOC) [file pone.0224266.s003.doc]

**Chochrane:**

#1 (C-reactive protein to Albumin ratio or C-reactive protein-to-Albumin ratio or C-reactive protein Albumin ratio)

#2 MeSH descriptor Carcinoma, Renal Cell explode all trees

#3 (renal OR kidney OR nephron*) NEAR (cancer* OR neoplasm* OR carcinoma* OR tumour* OR tumour*)

#4 (renal or kidney or nephron*) NEXT cell NEAR cancer*

#5 “clear cell type” NEXT/3 carcinom*

#6 (#2 OR #3 OR #4 OR #5)

#7 #1 and #6

**MEDLIME(Ovid SP):**

#1 (C-reactive protein to Albumin ratio or C-reactive protein-to-Albumin ratio or C-reactive protein Albumin ratio).mp

#2 exp Carcinoma, Renal Cell/

#3 ((renal OR kidney OR nephron$) adj6 (cancer$ OR neoplasms$ OR carcinoma$ OR tumour$ OR tumour$)).mp

#4 ((renal or kidney or nephron$) adj cell adj6 cancer$).mp

#5 ("clear cell type" adj3 carcinom$).mp

#6 or/2-5

#7 1 and 6

**Embase(Ovid SP):**

#1 (C-reactive protein to Albumin ratio or C-reactive protein-to-Albumin ratio or C-reactive protein Albumin ratio).mp

#2 exp kidney carcinoma/

#3 ((renal or kidney or nephron$) adj6 (cancer$ or neoplasms$ or carcinoma$ or tumour$ or tumour$)).mp.

#4 ((renal or kidney or nephron$) adj cell adj6 cancer$).mp.

#5 ("clear cell type" adj3 carcinom$).mp

#6 or/2-5

#7 1 and 6
